# Impact of Protein Nitration on Influenza Virus Infectivity and Immunogenicity

**DOI:** 10.1128/spectrum.01902-22

**Published:** 2022-10-31

**Authors:** Harrison Dulin, Nathan Hendricks, Duo Xu, Linfeng Gao, Keidy Wuang, Huiwang Ai, Rong Hai

**Affiliations:** a Cell, Molecular, and Developmental Biology Graduate Program, University of California, Riverside, Riverside, California, USA; b Microbiology and Plant Pathology, University of California, Riverside, Riverside, California, USA; c Proteomics Core, University of California, Riverside, Riverside, California, USA; d Department of Molecular Physiology and Biological Physics, University of Virginia, Charlottesville, Virginia, USA; Center for Research and Advanced Studies

**Keywords:** hemagglutinin, infection, influenza, lung infection, nitric oxide synthase, nitrotyrosine, peroxynitrite

## Abstract

Influenza viruses are deadly respiratory pathogens of special importance due to their long history of global pandemics. During influenza virus infections, the host responds by producing interferons, which activate interferon-stimulated genes (ISGs) inside target cells. One of these ISGs is inducible nitric oxide synthase (iNOS). iNOS produces nitric oxide (NO) from arginine and molecular oxygen inside the cell. NO can react with superoxide radicals to form reactive nitrogen species, principally peroxynitrite. While much work has been done studying the many roles of nitric oxide in influenza virus infections, the direct effect of peroxynitrite on influenza virus proteins has not been determined. Manipulations of NO, either by knocking out iNOS or chemically inhibiting NO, produced no change in virus titers in mouse models of influenza infection. However, peroxynitrite has a known antimicrobial effect on various bacteria and parasites, and the reason for its lack of antimicrobial effect on influenza virus titers *in vivo* remains unclear. Therefore, we wished to test the direct effect of nitration of influenza virus proteins. We examined the impact of nitration on virus infectivity, replication, and immunogenicity. We observed that the nitration of influenza A virus proteins decreased virus infectivity and replication *ex vivo*. We also determined that the nitration of influenza virus hemagglutinin protein can reduce antibody responses to native virus protein. However, our study also suggests that nitration of influenza virus proteins *in vivo* is likely not extensive enough to inhibit virus functions substantially. These findings will help clarify the role of peroxynitrite during influenza virus infections.

**IMPORTANCE** Nitric oxide and peroxynitrite produced during microbial infections have diverse and seemingly paradoxical functions. While nitration of lung tissue during influenza virus infection has been observed in both mice and humans, the direct effect of protein nitration on influenza viruses has remained elusive. We addressed the impact of nitration of influenza virus proteins on virus infectivity, replication, and immunogenicity. We observed that *ex vivo* nitration of influenza virus proteins reduced virus infectivity and immunogenicity. However, we did not detect nitration of influenza virus hemagglutinin protein in vivo. These results contribute to our understanding of the roles of nitric oxide and peroxynitrite in influenza virus infections.

## INTRODUCTION

Influenza virus infections remain a global health threat, responsible for seasonal epidemics and four major pandemics since the start of the last century, despite the availability of vaccines and antiviral medications. Influenza viruses are respiratory agents, infecting epithelial cells along the respiratory tract. Damage from virus infection can compromise lung function, which can prove fatal. The damage to the lungs during virus infection is due to a combination of direct cell death from virus infection and immune responses to the virus. One of the innate immune factors activated by virus infection is inducible nitric oxide synthase (iNOS), an interferon-stimulated gene product. Murine and human macrophages and neutrophils that infiltrate the lung during infection upregulate iNOS in response to interferon signaling, and iNOS in turn synthesizes nitric oxide (NO) from cellular l-arginine and molecular oxygen (1–5).

The roles of NO in microbial infection are diverse and seemingly paradoxical. NO acts as a signaling molecule through NO-sensitive guanylyl cyclase (NO-GC), which promotes vascular relaxation and pulmonary vasodilation, improving blood flow to the lungs and lung function (6, 7). NO has also been implicated in the modulation of immune responses during infection. iNOS knockout mice exhibit reduced proinflammatory cytokine profiles in the lung during infection compared to iNOS-competent mice (8). Additionally, NO is required for Th17 cell differentiation (9), and NO has been implicated in B-cell activation and plasma cell survival (10, 11). On the other hand, NO can react with superoxide radicals to form highly reactive nitrogen species (RNS), principally peroxynitrite (12, 13). Peroxynitrite and other radical species can react with macromolecules such as proteins, lipids, and nucleic acids. When reacting with proteins, RNS can oxidize serine residues and nitrate tyrosine residues to give nitrotyrosine, aminotyrosine, cysteine-sulfinic acid, cysteine-sulfonic acid, and *S*-nitrosothiol (14, 15). These modifications can alter protein function, and extensive nitration of cellular macromolecules can produce nitrative stress inside the cell. This damage to the cell has been linked to cellular stress and cell death (16).

Following influenza virus infection, protein nitration in the lungs is observed in both mice and humans by immunohistochemical staining for nitrotyrosine, indicating nitrative stress inside lung cells (17–19). This nitrative stress has been linked directly to the pathogenesis of influenza disease in mice. iNOS knockout mice have increased survival following infection with influenza virus compared to iNOS-competent mice (17, 20). This has also been observed with other pathogens, such as Sendai virus, Dengue virus, and Cryptococcus gattii (1, 21–24). The increased survival of iNOS knockout mice has been attributed to the reduced damage to the lung from reactive nitrogen species, principally peroxynitrite (1, 25). In addition to host macromolecules, peroxynitrite can also react with viral proteins, which can compromise protein function. Nitration of HIV reverse transcriptase *in vitro* and nitration of coxsackievirus protease 3C inhibits enzyme function through the nitration of amino acids in the enzyme active site (26, 27). Nitration of the severe acute respiratory syndrome coronavirus (SARS-CoV) Spike (S) protein reduces the cell-cell fusion activity mediated by S protein, which is associated with decreasing amounts of S protein palmitoylation (28). We hypothesized that nitration of influenza virus proteins could similarly alter protein function and, in turn, virus replication dynamics. However, more information is needed on the interaction between reactive nitrogen species and influenza virus proteins. The multiple roles of NO *in vivo* can possibly affect viruses in ways beyond direct nitration of virus proteins by peroxynitrite. We therefore wished to test if direct nitration of influenza virus proteins was sufficient to cripple virus infectivity and replication by *in vitro* nitration of influenza virions.

Additionally, posttranslational modifications to influenza virus proteins, such as glycosylation of hemagglutinin (HA), can affect antibody responses to HA (29, 30). Modification of B-cell epitopes can inhibit the recognition of virus antigen and reduce antibody responses to unmodified antigen. We hypothesized that nitration of HA could result in similar alterations in antibody responses. We therefore set out to examine the effects of nitration of influenza virus HA on antibody responses to HA protein. Finally, we sought to look for the *in vivo* nitration of influenza HA protein during infection. There have been many reports on the presence of nitrotyrosine, a marker for nitration, in the lung following influenza virus infection (17–19). However, these reports relied on immunohistochemical staining of the tissue for nitrotyrosine. Since peroxynitrite is a highly reactive nitrogen species that can react with either host or virus macromolecules, immunohistochemical staining of the lung tissue does not demonstrate direct nitration of virus macromolecules. To the best of our knowledge, this is the first report of experiments to detect the direct nitration of influenza virus proteins in infected lungs. We immunoprecipitated HA protein from mouse lung followed by mass spectrometry (mass spec, or MS) analysis to check for nitration of HA *in vivo.* In summary, we found that direct nitration of influenza virus *in vitro* had a deleterious effect on virus infectivity and replication dynamics. We also found that vaccination with nitrated HA protein reduced antibody responses to wild-type HA. However, although we detected immunoprecipitated HA protein from mouse lung, our mass spec analysis did not detect nitration of HA. We conclude with a discussion of the interplay between peroxynitrite, host macromolecules, and virus proteins.

## RESULTS

### *In vitro* nitration of hemagglutinin protein and influenza virions.

**FIG 1:**
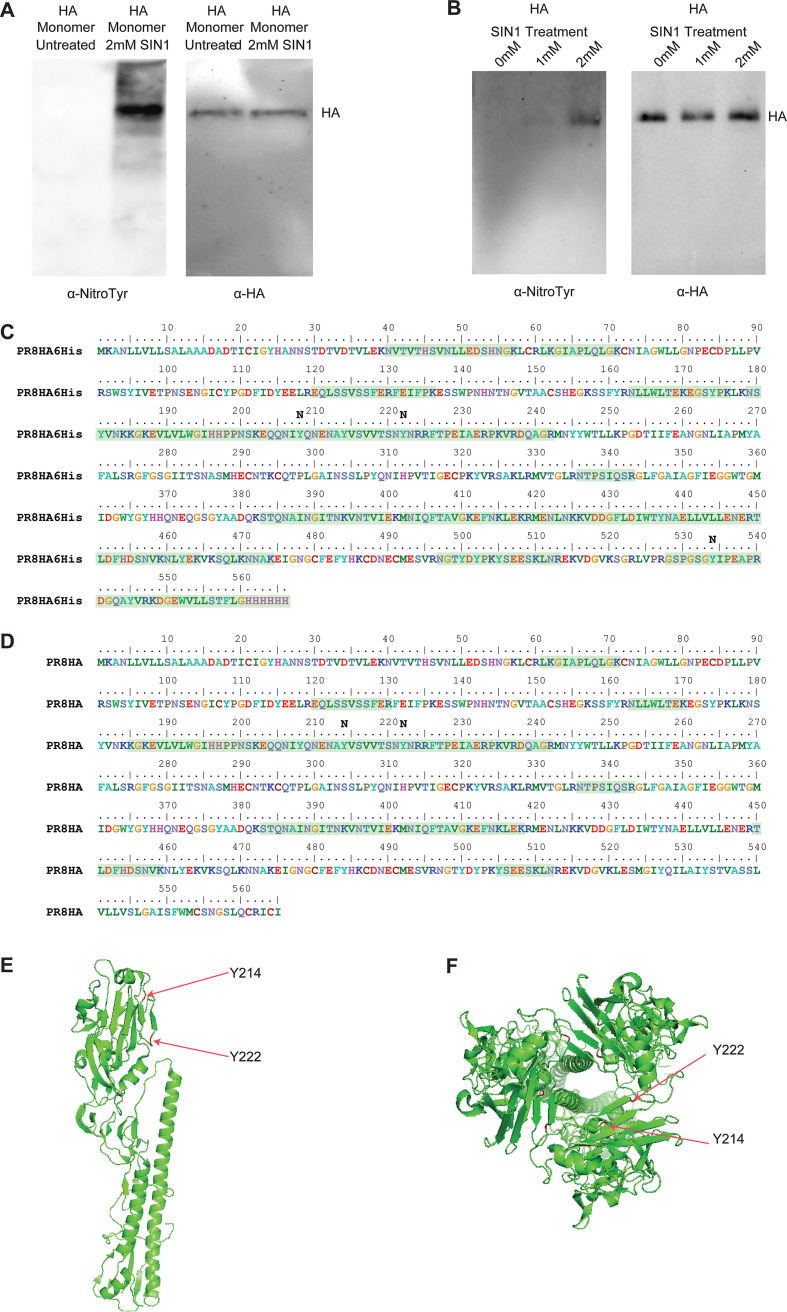
*In vitro* nitration of influenza HA protein and influenza virion with SIN-1. (A) Western blotting results, confirming nitrotyrosine modification of HA monomer. (B) Western blotting results confirming nitrotyrosine modification of influenza virion HA. (C) Mass spectrometry coverage and confirmation of nitrotyrosine modification of HA trimer (48.9% coverage). (D) Mass spectrometry coverage and confirmation of nitrotyrosine modification of influenza virion HA (26.6% coverage). Regions from detected peptides are highlighted in green. (E and F) Structural representations of the HA monomer (E) and trimer (F), showing the modified nitrotyrosine sites of panel D.

To understand the direct effects of nitration of influenza A virus proteins, we needed to separate the roles of NO as an innate immune response factor and signaling molecule from its role as a contributor to the formation of RNS. We therefore took an *in vitro* approach to directly nitrate influenza virus proteins. We used 3-morpholino-sydnonimine (SIN-1), which produces peroxynitrite under physiological conditions, to nitrate influenza virus proteins *in vitro* (31). We confirmed the presence of nitrotyrosine modification of HA by Western blotting (Fig. 1A and B) and mass spec analysis (Fig. 1C to F). The treated virions and proteins were used to study the effects of nitration on virus infectivity and the antibody response to virus proteins.

### SIN-1 treatment of influenza virus reduces virus infectivity and replication.

**FIG 2:**
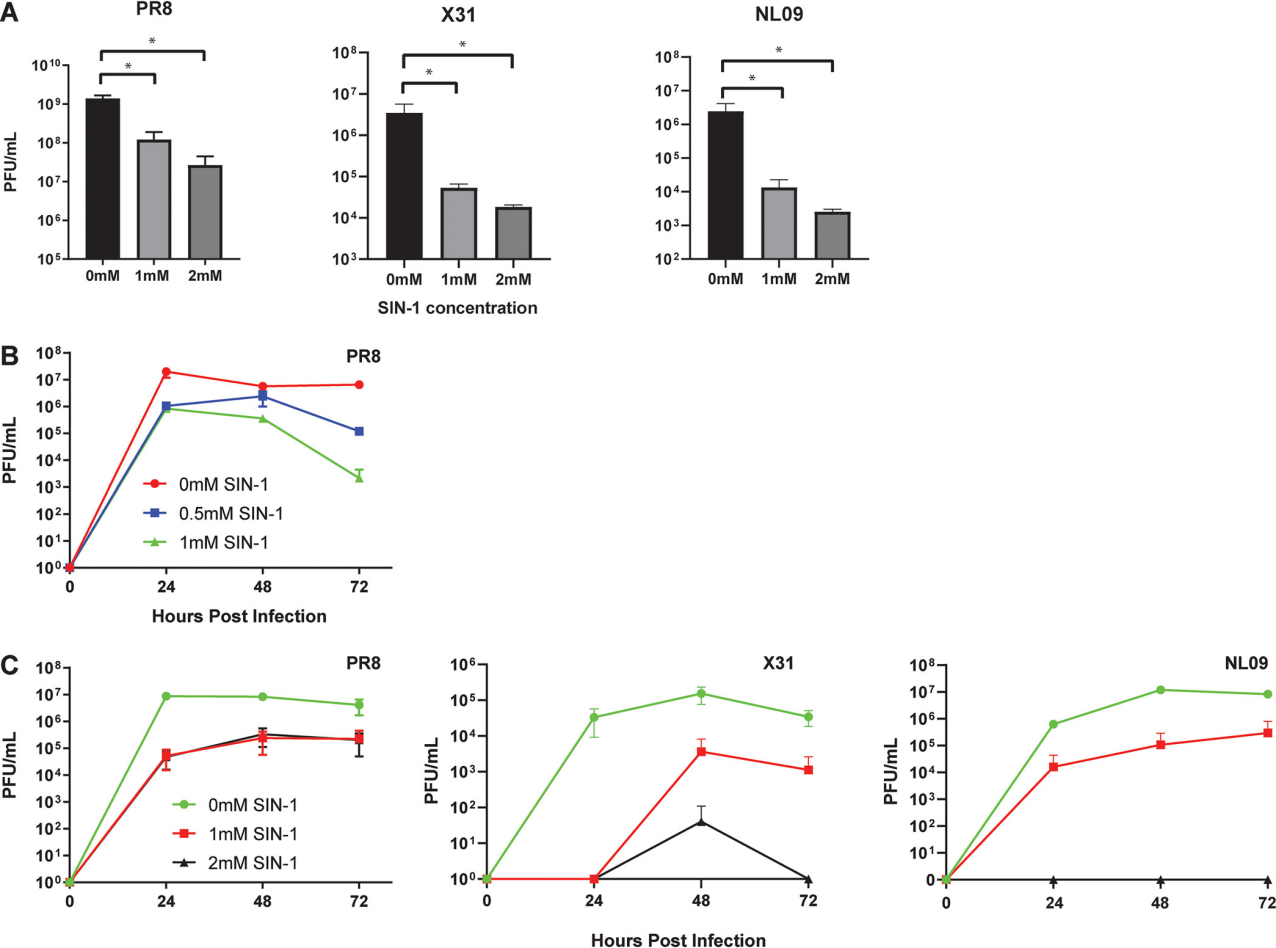
SIN-1 treatment of influenza virions. (A) Plaque assay titers of infectious virus following overnight SIN-1 treatment. *, *P* < 0.05; statistical significance was determined by two-tailed unpaired *t* test. (B) Growth curve of PR8 virus with SIN-1 included in growth medium. (C) Growth curves of influenza viruses pretreated with SIN-1.

**FIG 3:**
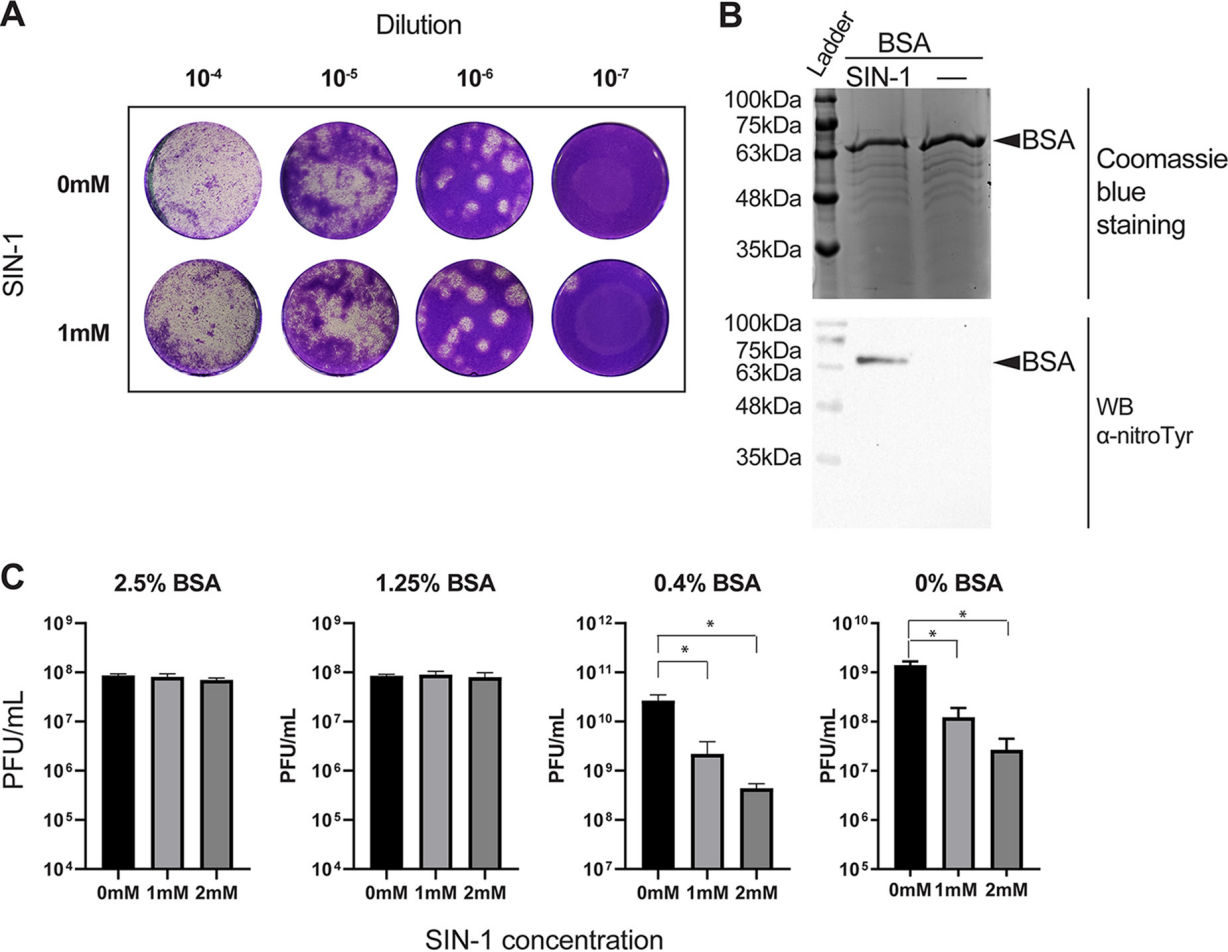
SIN-1 treatment of PR8 influenza virus in the presence of BSA. (A) Representative plaques of PR8 influenza virus following SIN-1 treatment in the presence of 2.5% BSA. (B) Western blot of SIN-1-treated BSA to detect the presence of nitrated BSA. (C) Infectivity of SIN-1-treated PR8 virus in the presence of different concentrations of BSA. *, *P* < 0.05; statistical significance was determined by two-tailed unpaired *t* test. Each graph shows results from a separately performed experiment.

Mass spec analysis of the SIN-1-treated influenza virus revealed nitration of tyrosine residues at positions Y214 and Y222, which are in the receptor-binding domain of HA (32) (Fig. 1C). We hypothesized that nitration of the receptor-binding domain might reduce influenza virus infectivity and hinder virus replication. To determine the effect of SIN-1 treatment on the ability of influenza viruses to infect cells, strains PR8, NL09, and X31 of purified influenza virus were treated overnight with either 0 mM, 1 mM, or 2 mM SIN-1 in phosphate-buffered saline (PBS). Infectious virus concentrations were determined by a standard plaque assay analysis. SIN-1 treatment in PBS resulted in significantly fewer plaques compared to untreated virus (Fig. 2A). To further study the effect of SIN-1 treatment on virus infectivity and replication, we performed influenza virus growth curves in the presence of SIN-1. SIN-1 significantly reduced the replication of influenza virus in MDCK cells (*P* < 0.05) (Fig. 2B). Furthermore, we performed a growth curve with influenza viruses pretreated with SIN-1. PR8, NL09, and X31 were treated overnight with 2 mM or 1 mM SIN-1 and then allowed to infect MDCK cells for the virus growth curve. The preinfection SIN-1 treatment gave significantly reduced virus titers (*P* < 0.05) (Fig. 2C). To better mimic *in vivo* nitration conditions, which include macromolecules other than virus proteins, we treated PR8 virus with SIN-1 in the presence of increasing amounts of bovine serum albumin (BSA), which acted as a nonviral target for nitration. SIN-1 treatment in the presence of 1.25% or 2.5% BSA resulted in no change in plaque titers compared to untreated virus (Fig. 3). This supported the notion that nitration of influenza viruses reduces virus infectivity and replication, but this effect may be mitigated by the presence of other host macromolecules.

### SIN-1 treatment of HA protein lowers virus-specific antibody responses.

**FIG 4:**
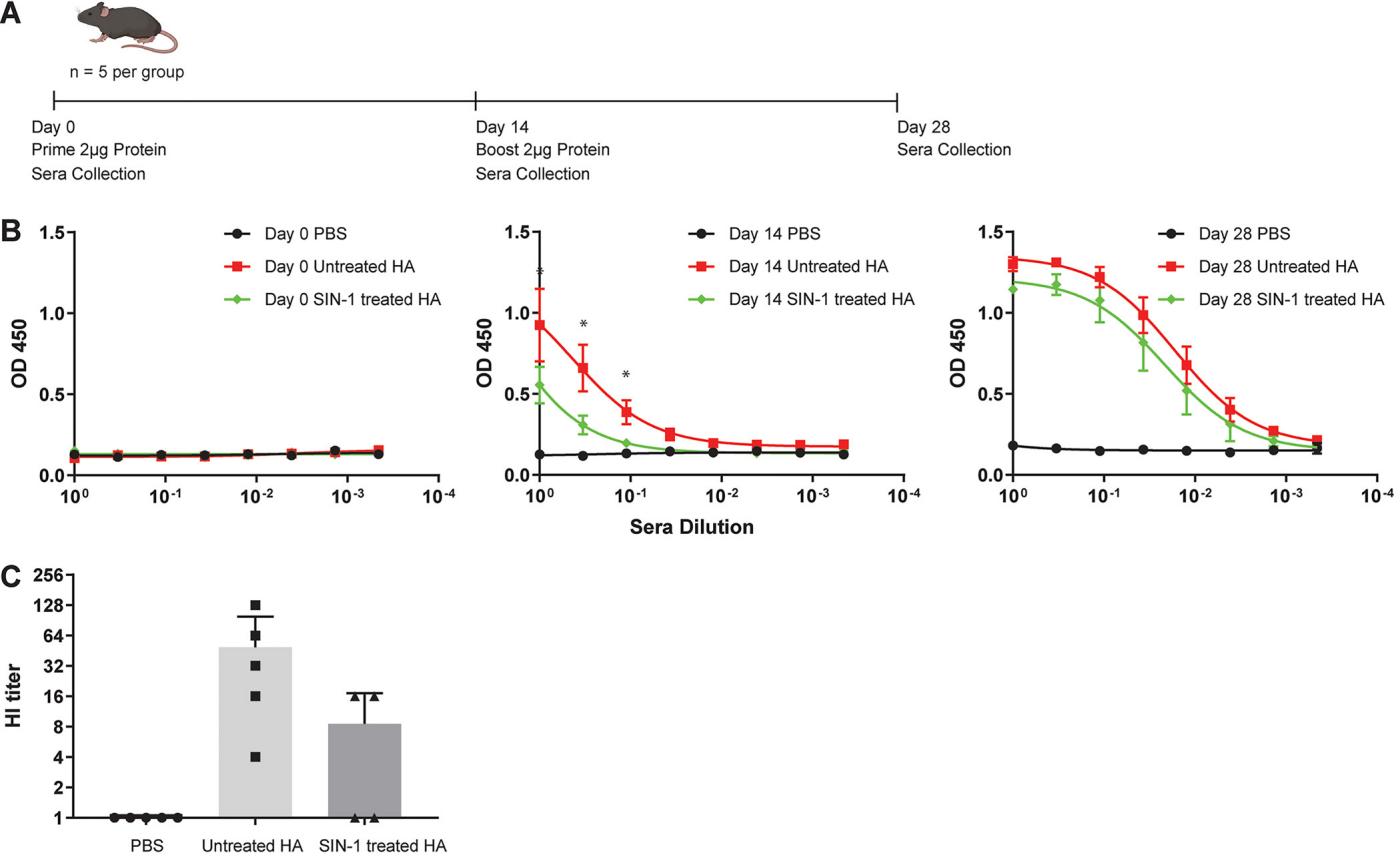
Reduced antibody responses to SIN-1-treated HA protein. (A) Schedule of mouse vaccinations with SIN-1-treated or untreated HA protein, and of sera collection (*n* = 5 per group). (B) ELISA for mouse sera following vaccination with SIN-1-treated HA protein. *, *P* < 0.05, unpaired *t* test of significance for untreated versus 20-h SIN-1-treated HA for all dilutions on day 14. (C) Hemagglutination inhibition assay results with sera from day 28 postvaccination.

**FIG 5:**
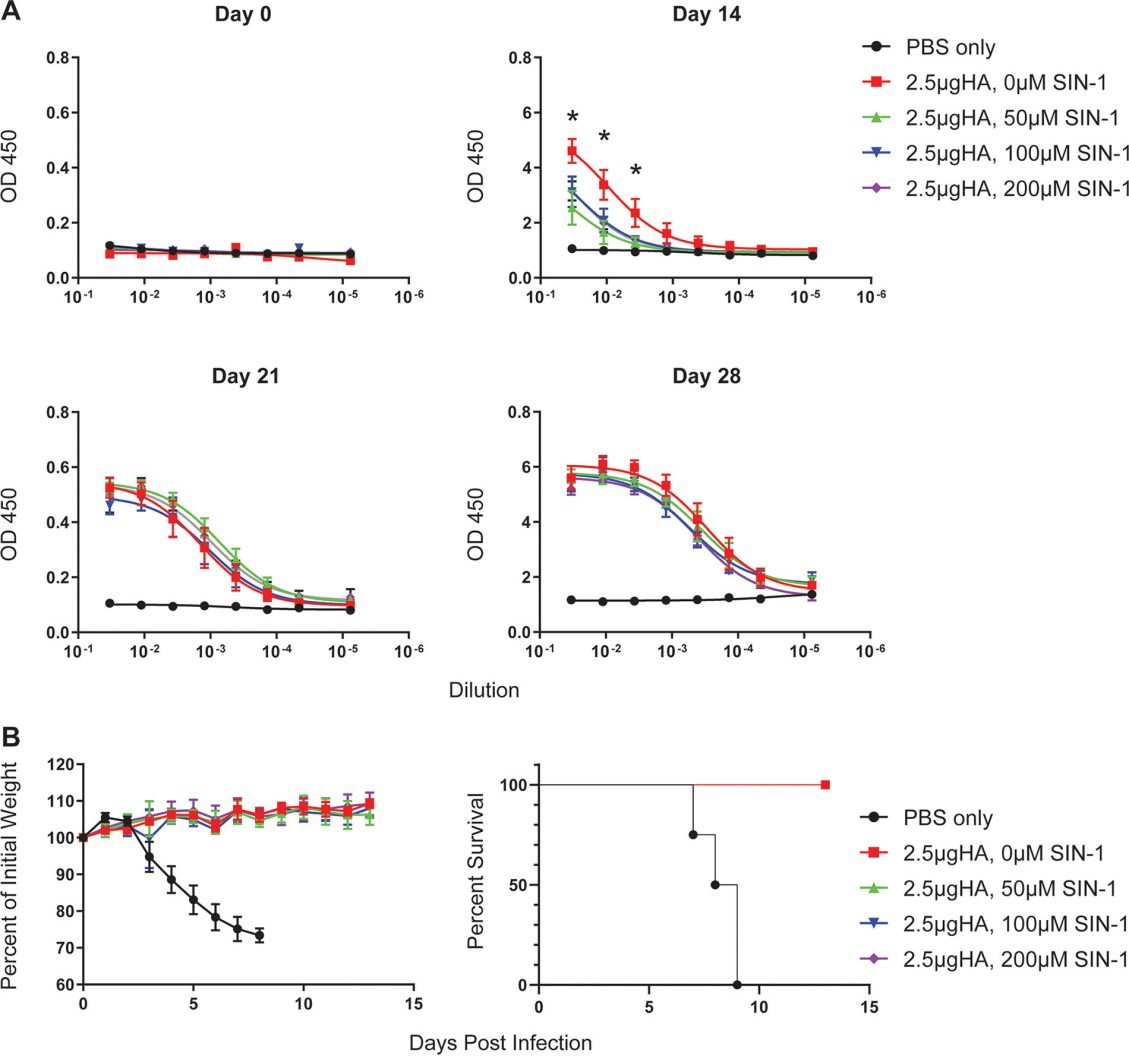
Mouse responses to vaccination with SIN-1-treated HA. (A) Mice (*n* = 5 per group) were vaccinated with 2.5 μg of SIN-1-treated HA and boosted on days 14 and 21 postvaccination. Sera were collected on days 0, 14, 21, and 28 postvaccination. Antibody responses to influenza virion were determined by ELISA. (B) Weight loss and percent survival of the mice from the groups in panel A, following challenge with a lethal dose of PR8.

The head domain of the PR8 influenza HA protein contains 5 immunodominant antigenic regions: Sa, Sb, Ca_1_, Ca_2_, and Cb (33). Our mass spec analysis of the SIN-1-treated purified HA protein showed nitration of tyrosine residue Y208, which is within the Sb antigenic region (Fig. 1C). We therefore hypothesized that nitration of influenza virus proteins could alter viral epitopes and reduce antibody responses to native virus proteins. To test this hypothesis, C57BL6 mice were vaccinated with 2 μg of purified trimeric HA protein treated with 0.2 mM SIN-1 for different lengths of time. Mice were boosted with an additional 2 μg of protein at 2 weeks postvaccination (Fig. 4A). Sera were collected on day 0, day 14, and day 28 after the first vaccination. An enzyme-linked immunosorbent assay (ELISA) was performed with plated influenza virions to determine antibody responses following vaccination with HA protein. We observed decreased antibody responses with increased SIN-1 treatment (see Fig. S1 in the supplemental material), with a significant decrease in antibody responses observed after 20 hours of treatment with SIN-1 (Fig. 4B). The decrease was most pronounced following the first vaccination. After boosting, antibody titers remained lower in the mice given SIN-1-treated HA, but only slightly. Additionally, significantly reduced antibody responses to HA were observed when purified HA protein was treated with different concentrations of SIN-1 for 1 hour at room temperature (*P *< 0.05) (Fig. 5A). After boosting, the differences in antibody titers between the groups were not significant, and all the mice survived challenge with a lethal dose of parental PR8 virus (Fig. 5B). Because our mass spec data identified nitration of tyrosine residues in the receptor-binding domain of HA (Fig. 1), we further hypothesized that SIN-1 treatment of HA would result in decreased virus-neutralizing antibodies. We used sera from day 28 postvaccination to determine microneutralization antibody titers from mice vaccinated with SIN-1-treated or untreated HA protein. While the sera from mice vaccinated with the SIN-1-treated HA protein had reduced microneutralization titers compared to mice vaccinated with untreated HA protein, the difference was not statistically significant (*P* = 0.14) (Fig. 4C). In summary, *in vitro* nitration of influenza virus HA protein is associated with decreased antibody responses to HA.

### *In vivo* nitration of HA.

**FIG 6:**
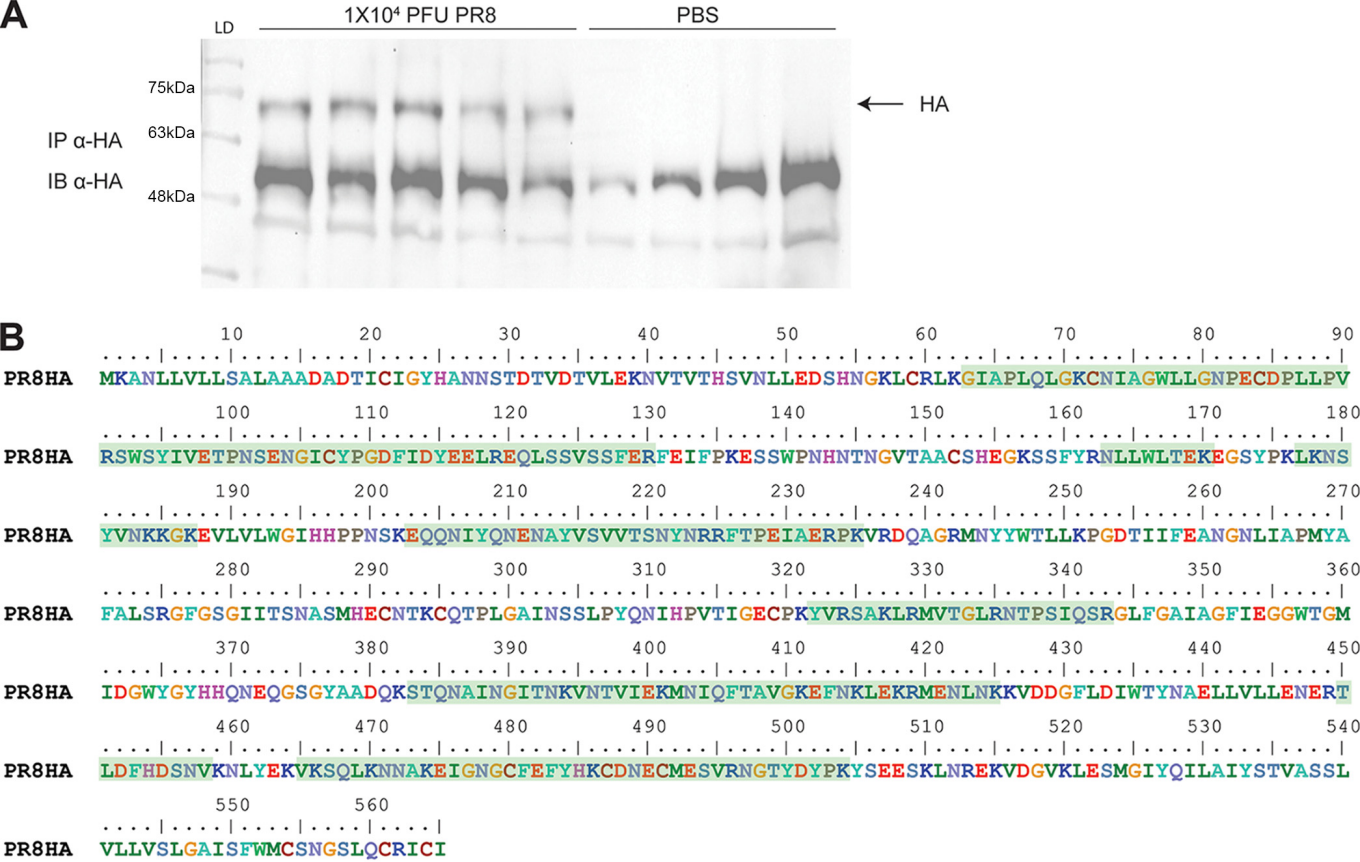
Immunoprecipitation and mass spectrometry analysis of HA protein from mouse lung. (A) Western blot for the detection of HA protein from mouse immunoprecipitations. Protein was immunoprecipitated with human α-HA antibody and blotted with mouse α-HA. Wells from left to right: ladder, samples from 5 mice infected with 10^4^ PFU of PR8 virus, samples from 4 uninfected mouse controls. (B) Coverage of HA protein mass spec analysis. Regions from detected peptides are highlighted in green. (Protein accession number A0A6H1QXP1; 41.6% coverage, 24 unique peptides).

To determine if influenza hemagglutinin protein is nitrated during influenza virus infection, we immunoprecipitated HA protein from the lungs of mice infected with 10,000 PFU of influenza A PR8 virus at day 5 postinfection. The immunoprecipitated protein was detected by Western blotting (Fig. 6A). The band corresponding to the HA protein was excised from the gel and used for mass spec analysis for the detection of the following RNS-induced modifications: nitrotyrosine, aminotyrosine, cysteine-sulfinic acid, cysteine-sulfonic acid, and *S*-nitrosothiol. Detected peptides were checked against a mouse peptide library as a negative control. While we were able to detect the presence of the HA protein, nitrotyrosine modification of HA was not detected, nor were additional RNS modifications (Fig. 6B). To check if host proteins were nitrated to nitrotyrosine following infection, the immunoprecipitated samples from Fig. 6A were analyzed by mass spec against a mouse database. We were able to detect nitration of several different proteins, including actin protein (Fig. S3).

## DISCUSSION

We observed that influenza virus proteins are susceptible to nitration by peroxynitrite *in vitro*. Nitration modifications to the HA protein can occur in important immunological domains and in the receptor-binding domain. Nitration of influenza viruses reduces virus infectivity, resulting in lower virus titers *ex vivo*. Nitration of HA can also reduce titers of HA-specific antibodies. While influenza virus is susceptible to nitration, nitration may not occur extensively enough to significantly impact virus titers or virus immunogenicity *in vivo*.

The decreased ability of influenza viruses to infect cells following SIN-1 treatment shows that, in principle, RNS modifications to influenza virus proteins can have a negative impact on virus infectivity and replication. However, the question remains as to whether these modifications happen extensively during *in vivo* infection. Blocking NO in a mouse model of influenza virus infection had no effect on virus titers in mouse lungs (17, 34). Similarly, inhaled NO did not affect virus titers in mouse lungs (35). These findings suggest that the effects of NO during infection are not a result of direct interaction with virus proteins. Our mass spec analysis of immunoprecipitated HA protein found no evidence of ROS- or RNS-induced modifications to the HA protein. This did not rule out the possibility that influenza virus proteins may be modified *in vivo*. Nitrotyrosine can be detected in mouse lungs as early as day 3 postinfection with influenza virus (18, 36). However, Akaike et al. reported peak NO production in mouse lungs on day 7 postinfection, and peak nitrotyrosine levels were typically seen around days 7 and 8 postinfection (17, 18, 37, 38). We collected the mouse lungs on day 5 postinfection. This earlier time point was chosen to maximize the amount of HA recovered from immunoprecipitation. A later time point may show nitration of the HA protein, but it is more challenging to perform this experiment as the virus starts to be cleared from the lungs and the mice may succumb to the virus before reaching the desired day postinfection. It may also be that the HA protein is modified below the limit of detection of our mass spec analysis. Additionally, we only checked the HA protein for modification, but other virus proteins may be more susceptible to nitration. However, the lack of RNS modifications to HA from our immunoprecipitation of HA from mouse lungs suggested that RNS modifications to influenza virus proteins may not occur to a significant extent *in vivo*.

One explanation for why influenza virus proteins may not be modified *in vivo* is that *in vivo* concentrations of peroxynitrite may be lower than the concentrations we used *in vitro*. While the transient and highly reactive nature of peroxynitrite makes determining its concentration *in vivo* challenging, estimates have suggested *in vivo* rates of peroxynitrite production around 5 μM s^−1^, which is difficult to convert to the actual concentrations of peroxynitrite used in our *in vitro* experiments (16, 23, 39, 40). Another explanation is that peroxynitrite is far more likely to contact and react with host molecules than viral molecules. During influenza virus infection, virion mass makes up only about 1% of the total dry mass of the cell (41, 42). Stoichiometrically, if there are more host proteins for the peroxynitrite to react with than viral proteins, modifications to viral proteins will be reduced, and differences in virus titers will not be significant. This possibility was supported by our measurement of virus infectivity following SIN-1 treatment in the presence or absence of BSA. BSA is known to undergo nitration to nitrotyrosine following SIN-1 treatment, and SIN-1 treatment of influenza virus in the presence of excess BSA did not affect virus infectivity (Fig. 3). This suggested that when other macromolecules are present to act as targets for RNS modification, modifications of virus molecules are reduced, and virus infectivity and replication are unimpeded. This suggestion is somewhat at odds with the growth curve results we obtained with infected cells in the presence of SIN-1 (Fig. 2B). We observed a decrease in virus titers in the presence of SIN-1 in virus-infected cells when an abundance of host proteins was also present. While this might suggest a direct role for peroxynitrite in inhibiting influenza virus replication, it could also be the result of SIN-1-induced pH changes in the culture medium or direct damage to the cells from the peroxynitrite. While influenza virus growth curves are typically done in our lab with medium containing buffers such as HEPES, HEPES can interfere with peroxynitrite production from SIN-1, and so it was not included in our growth curve medium (43, 44). This resulted in lower pH in SIN-1-treated wells than in untreated wells at 72 hours postinfection, and this lower pH could account for the lower viral titers in the growth curves.

The decreased antibody responses we observed to SIN-1-treated HA protein showed that, in principle, RNS-induced modifications to virus proteins can reduce antibody responses to native virus. However, the reduced antibody responses were seen principally following the first vaccination. Boosting the mice resulted in no differences in antibody responses between mice vaccinated with SIN-1-treated HA and mice vaccinated with untreated HA. One possible explanation for this is that the nitration of the HA protein occurs randomly at different side amino acid changes. In this case, the vaccinations include a mixture of HA proteins modified at different positions. While the initial vaccination may include enough modifications to reduce antibody responses to native virus, because the modifications occur randomly, there is no guarantee that boosting would contain the same mixture of modified epitopes as the original vaccination. There is also no guarantee that each mouse receives the same mixture of modified epitopes. Boosting would then preferentially increase antibody responses to unmodified epitopes. This could explain why antibody responses in the mice vaccinated with SIN-1-treated HA were more similar to wild-type responses after the boost. This also raised the question whether RNS-induced modifications to virus proteins *in vivo* are extensive enough to significantly alter antibody responses. The conclusions reached in the previous paragraph also apply to our results obtained from vaccinating with the SIN-1-treated HA protein. While in principle RNS modifications may reduce antibody responses to HA proteins, modifications likely do not occur extensively enough to make a significant difference to antibody responses *in vivo*. Peak nitrotyrosine production may occur at a point when virus protein levels are already in decline, which would suggest that most virus protein produced is not modified extensively *in vivo.* However, as iNOS has roles in immune signaling, the NO and peroxynitrite may still impact antiviral antibody levels indirectly. Jayasekera et al. reported increased IgG2a antibody responses in mice lacking iNOS, but they attributed this to altered interferon levels and T-cell profiles in the iNOS knockout mice, rather than to a loss of nitration of influenza virus proteins (8). These indirect effects may have a more significant role in determining antibody titers than direct nitration of virus proteins.

While levels of peroxynitrite in healthy animals are low, preexisting inflammatory conditions may elevate levels of peroxynitrite. Circulating levels of peroxynitrite in sera are increased in animal models of hypertension, and iNOS expression is increased in the lungs of mice acutely exposed to cigarette smoke (14, 45, 46). It is possible that exposure to influenza virus proteins from either infection or vaccination under conditions with high preexisting levels of peroxynitrite may increase the likelihood of nitration of influenza virus proteins, which may lead to some of the effects of nitration observed in this study, such as lower antibody responses.

In summary, influenza viruses are susceptible to protein nitration, and *in vitro* nitration of influenza virus proteins reduces virus infectivity and immunogenicity. However, nitration of virus proteins may not occur extensively *in vivo* in previously healthy animals. The impact of direct protein nitration in other pathogenic virus infections remains an area of ongoing research.

## MATERIALS AND METHODS

### Influenza virus strains and purification of influenza virions.

Growth curves following SIN-1 (Cayman Chemicals) treatment were performed with PR8 A/Puerto Rico/8/34(H1N1) virus, X31 mouse-adapted H3N2 reassortant virus carrying the HA and NA genes of A/Hong Kong/1/1968 (H3N2) in the background of PR8, and NL09 A/Netherlands/602/2009 (H1N1) virus. All other experiments were performed with PR8 virus. Ten-day-old embryonated chicken eggs were infected with 50 PFU of virus per egg in 100 μL of PBS–BSA–penicillin-streptomycin (pen-strep) as previously described and incubated at 37°C for 48 h (47, 48). Eggs were then kept at 4°C overnight, and the allantoic fluid was collected. The allantoic fluid was spun down at 3,000 rpm for 30 min at 4°C, and the supernatant was collected. Virion was purified from allantoic supernatant by 20% sucrose–NTE buffer ultracentrifugation containing 100 mM NaCl, 10 mM Tris-HCl, and 1 mM EDTA at 25,000 rpm for 2 h at 4°C. The resulting pellets were resuspended in 1 mL PBS, and virion concentration was determined by Bradford assay.

### Purification of HA protein.

Ten T175 flasks (Genesee Scientific) of 293T cells were polyethylenimine transfected with 20 μg of pCAGGS expression plasmid containing the C-terminal trimerization domain and C-terminal 6×His-tagged PR8 HA lacking the intracellular and transmembrane domains. Cells were transfected in 10 mL Opti-MEM medium. At 12 h after transfection, the medium was changed to 20 mL Dulbecco’s modified Eagle’s medium containing 10% FBS, and cells were left at 37°C for 48 h. After 48 h, the supernatant was collected, and protein was purified with a 5-mL HisTrap column (Cytiva). Eluted protein was diafiltrated with a 10-kDa-cutoff column (Amicon Ultracel 10K centrifugal filters) and washed twice with PBS. Protein concentration was determined by Bradford assay, and trimeric HA was separated from monomeric HA by size exclusion chromatography.

### *In vitro* nitration of influenza proteins and BSA with SIN-1 chloride.

For the SIN-1 treatment of HA monomer (Fig. 1), 9 μg of purified HA monomer was added to PBS containing 200 μM SIN-1 in a 125-μL total volume and left shaking overnight at 4°C. For the SIN-1 treatment of BSA (Fig. 3), 12 μg of BSA was added to PBS containing 2 mM SIN-1 in a 125-μL total volume and left shaking overnight at 4°C. For the SIN-1 treatment of HA trimeric protein for mouse vaccinations, 100 μL of HA trimer (93 μg) was added to PBS containing SIN-1 in a final volume of 500 μL. Samples were left overnight at 4°C and then diafiltrated with a 10-kDa-cutoff column (Amicon Ultracel 10K centrifugal filters) and washed twice with 5 mL PBS to remove residual SIN-1 in solution. The protein concentration was then determined by Bradford assay.

### Coomassie staining and Western blotting.

Western blots for HA protein were blocked with 5% milk in PBS containing 0.1% Tween 20 and then blotted with PY102 HA mouse monoclonal antibody diluted 1:500 in blocking buffer. The antibody was a kind gift from Peter Palese at the Icahn School of Medicine at Mount Sinai. When performing Western blot assays to detect nitrotyrosine, samples were added to 2× SDS-PAGE loading dye lacking beta-mercaptoethanol or other reducing agents to avoid reducing nitrotyrosine to aminotyrosine. Western blots were blocked with 2% BSA in PBS containing 0.1% Tween 20 and then incubated with nitrotyrosine mouse monoclonal antibody (Cayman Chemicals) diluted 1:500 in blocking buffer. Blots were incubated with goat α-mouse IgG–horseradish peroxidase (HRP) secondary antibody (Prometheus) diluted 1:10,000 in blocking buffer. Blots were visualized with a Bio-Rad ChemiDoc Touch imaging system. Coomassie gels were stained as previously described (49).

### Mass spectrometry sample preparation.

For SIN-1-treated virus and protein samples, vacuum-dried samples were resuspended in 50 μL 100 mM triethylammonium bicarbonate (TEAB; Sigma-Aldrich, St. Louis, MO). Samples were reduced with the addition of 2.5 μL of 500 mM Tris(2-carboxyethyl)phosphine (Thermo Scientific, Rockford, IL) and incubated at 37°C for 1 h, after which 3 μL of 500 mM iodoacetamide (Sigma-Aldrich) was added. Samples were incubated in the dark at room temperature for 1 h. Samples were diluted with the addition of 250 μL of water and 250 μL of 100 mM TEAB. One microliter (200 ng) of trypsin-LysC mix (Promega, Madison, WI) was added to the samples, and the samples were digested at 37°C for 16 h. Ten microliters of the digest was injected for liquid chromatography (LC)-MS analysis.

For immunoprecipitation samples, bands corresponding to the size of full-length HA protein were cut from the gel, and in-gel digestion was performed by adding 500 μL of 25% acetonitrile–50 mM ammonium bicarbonate (ABC) for 10 min. Samples were sonicated for 15 min, and then the solution was discarded and replaced with 500 μL of 50% acetonitrile–50 mM ABC. Samples were sonicated again for 15 min, and then solution was discarded and replaced with 500 μL of 25% acetonitrile–50 mM ABC. Samples were sonicated again for 15 min, and then the solution was discarded and replaced with 500 μL of 50% acetonitrile–50 mM ABC. Samples were sonicated again for 15 min, and then the solution was discarded and replaced with 500 μL of 100% acetonitrile. Samples were sonicated for 10 min, and then the solution was dried using a SpeedVac. Samples were reduced with the addition of 400 μL of 10 mM dithiothreitol–50 mM ABC and incubated at 37°C for 1 h, after which 49 μL of 500 mM iodoacetamide was added. Samples were incubated in the dark at room temperature for 25 min. Samples were washed twice with 500 μL of 100% acetonitrile with 5-min sonication, then dried using a SpeedVac. Samples were then suspended in 100 μL of trypsin solution and incubated at 37°C overnight. A 500-μL volume of 25% acetonitrile–5% hydrogen acetate (HAc) was then added to the trypsin-digested samples, and samples were sonicated for 20 min. An additional 300 μL of 50% acetonitrile–5% HAc was added to the samples, and samples were sonicated for 20 min. The solution was then dried using a SpeedVac and desalted using a C_18_ zip-tip (Waters). The peptide solution was then dried by SpeedVac and stored at −80°C until liquid chromatography-tandem mass spec analysis.

### Mass spectrometry sample and data analysis.

For SIN-1-treated virus and protein samples, LC was performed on a Waters nanoAcquity UPLC in single-pump trapping mode with a Thermo PepMap RSLC C_18_ Easy-spray column (2 μm, 100 Å, 75 μm by 25 cm) and a Waters Symmetry C_18_ trap column (5 μm, 100 Å, 180 μm by 20 mm). Solvents used were the following: A, water with 0.1% formic acid; B, acetonitrile with 0.1% formic acid. Samples were separated at 300 nL/min with a 260-min gradient starting at 3% B, increasing to 30% B from 1 to 230 min, then to 85% B at 240 min and a hold for 10 min, then back to 3% B in 10 min. Mass spectrometry data were acquired on a Thermo Orbitrap Fusion system in data-dependent mode. A full scan was conducted using 60k resolution in the Orbitrap in positive mode. Precursors for MS/MS were filtered by monoisotopic peak determination for peptides (set to small molecules for the analysis), an intensity threshold of 5.0e3, charge state of 2 to 7, and 60-s dynamic exclusion after 1 analysis, with a mass tolerance of 10 ppm. Collision-induced dissociation spectra were collected by MS/MS at 35% energy and an isolation window of 1.6 *m/z*. Results were searched individually in Proteome Discoverer 2.2 (Thermo Scientific) against a custom FASTA database with the His-tagged hemagglutinin sequence for purified protein samples. The precursor mass tolerance was set to 10 ppm, and fragment mass tolerance was set to 0.6 Da. Fixed modifications were carbamidomethyl (Cys, +57.021 Da), and dynamic modifications included methionine oxidation (+15.995 Da), N-terminal acetylation (+42.011 Da), and tyrosine nitration. Results were filtered to a strict 1% false-discovery rate.

For HA immunoprecipitation samples, liquid chromatography was performed on a Dionex Ultimate 3000 UPLC system. First, the peptide samples were loaded onto a precolumn (75-μm internal diameter [ID], 4-cm length) packed in-house with reversed-phase C_18_ material (ODS-AQ C18; 5-μm particle size, Dr. Maisch GmbH HPLC). The analytes were subsequently resolved on an analytical column (75 μm ID, 25 cm in length) packed with reversed-phase C_18_ material (ReproSil-Pur 120 C18-AQ, 3-μm particle size, Dr. Maisch GmbH HPLC). Solvents used were as follows: A, water with 0.1% formic acid; B, acetonitrile with 0.1% formic acid. Samples were separated at 300 nL/min with a gradient that entailed the following steps: 0 to 15 min, 95% A; 15 to 150 min, 95 to 63% A; 150 to 152 min, 63% to 1% A; 152 to 179 min, 1% A; 179 to 179.01 min, 1% to 95% A; 179.01 to 200 min, 95% A. Mass spectrometry data were acquired on a Q Exactive Plus Quadrupole-Orbitrap mass spectrometer (Thermo Fisher Scientific) in data-dependent mode, where one full-scan MS (*m/z* 300 to 2000) was followed by MS/MS scans on the 20 most abundant ions found with full-scan MS. Precursor ions were isolated at a width of 1.0 *m/z* unit, and dynamic exclusion was enabled with an exclusion time window of 60 s after a precursor ion was first selected for MS/MS acquisition. An intensity threshold of 5.0e3 was used, with charge state of 2 to 7 and 60-s dynamic exclusion after 1 analysis with a mass tolerance of 10 ppm. Collisionally induced dissociation spectra were collected in MS/MS at 35% energy and isolation window 1.6 *m/z*. The raw data were processed and analyzed using MaxQuant (version 2.0.3.1) against a custom FASTA database with the hemagglutinin sequence for the PR8 HA sequence. The precursor mass tolerance was set to 10 ppm and fragment mass tolerance was set to 0.6 Da. Fixed modification was carbamidomethyl (Cys +57.021 Da), and dynamic modifications included methionine oxidation (+15.995 Da), N-terminal acetylation (+42.011 Da), and tyrosine nitration. Results were filtered to a strict 1% false-discovery rate.

### Virus plaque assays.

Influenza virus plaque assays were performed as previously described (50). Influenza viruses were serially diluted 10-fold in PBS-BSA containing 1% Pen-Strep. A 250-μL volume of virus dilution was added to a confluent monolayer of MDCK cells in a 12-well plate, and virus was allowed to infect cells for 1 h at 37°C. Virus dilutions were replaced with posttransfection medium containing 3.7% Avicel RC-591 NF (FMC Corporation) and left at 37°C for 48 h. Cells were fixed with 1 mL of 3.7% paraformaldehyde for 1 h at room temperature and stained with 1% crystal violet solution.

### Virus infectivity following SIN-1 treatment.

Purified influenza virion was diluted to 2.75 ng/μL in a 125-μL total volume in PBS with SIN-1. Samples were left shaking at 350 rpm overnight at 4°C. After incubation, virus infectivity was determined by plaque assay. For SIN-1 treatment of virus in the presence of BSA, purified influenza virus was diluted to 2.75 ng/μL in a 120-μL total volume of PBS containing different concentrations of BSA, followed by the addition of 5 μL of SIN-1 to the indicated final concentrations. Samples were left shaking at 350 rpm overnight at 4°C. After incubation, virus infectivity was determined by plaque assay.

### Growth curves for influenza virus.

For growth curves following SIN-1 treatment, virus stocks were diluted to 2 × 10^3^ PFU/mL in a 1-mL total volume. Next, 200 μL of SIN-1 in PBS was added to the final concentrations of SIN-1 specified in the Fig. 2 legend and then incubated overnight at 4°C with shaking. Aliquots of 250 μL of each virus sample were used to infect monolayers of MDCK cells in a 12-well plate. After allowing virus to infect the monolayer for 1 h, medium was changed to 1 mL postinfection medium containing 0.5 μg trypsin-tosylsulfonyl phenylalanyl chloromethyl ketone (TPCK). One hundred microliters of supernatant was collected at 24, 48, and 72 h postinfection and replaced with 100 μL fresh postinfection medium. Virus titer at each time point postinfection was determined by plaque assay. For growth curves in the presence of SIN-1, MDCK cells were infected with 500 PFU of PR8 virus in a 12-well plate. Virus was allowed to infect cells for 1 h, then medium was changed to 1 mL of posttransfection medium containing 0.5 μg trypsin-TPCK with SIN-1. Sixty microliters of fresh SIN-1 in PBS was added every 12 h to restore initial SIN-1 concentrations, and 120 μL of medium was collected at 24, 48, and 72 h postinfection. Virus titer at each time point postinfection was determined by plaque assay.

### Mouse vaccinations and virus challenges.

Six-week-old female C57BL/6 mice were purchased from Jackson Laboratory and used for vaccination and challenge studies. SIN-1-treated influenza HA protein vaccine was diluted in PBS and added to an equal volume of Addavax adjuvant. Mice were injected intramuscularly with 50 μL of vaccine per injection in both back legs. Retro-orbital blood was collected and centrifuged for 10 min at 10,000 × *g* to separate out sera. Sera was collected and stored at −80°C for use in antibody studies. For the challenge study, mice were anesthetized with isoflurane and challenged intranasally with a lethal dose of 1,000 PFU of PR8 virus in 50 μL of solution. Mice were monitored for weight loss and mortality each day postinfection for 14 days.

### Mouse lung cell preparation and immunoprecipitation of HA protein.

Mice were challenged with 10^4^ PFU of PR8 virus and sacrificed humanely on day 5 postinfection. Lungs were collected and added to 10 mL of fluorescence-activated cell sorter (FACS) buffer (PBS, 2 mM EDTA, 3% FBS). Lungs were macerated until pulp-like with razor blades and added to 3 mL of lung digestion buffer (Hanks balanced salt solution [Lonza] containing 5% FBS, 1 mg/mL collagenase A, and 0.05 mg/mL DNase I). Lungs were digested for 30 min at 37°C, then passed through an 18-gauge needle to break up remaining tissue. The digested cells were added to 10 mL of fresh FACS buffer and spun down for 4 min at 400 × *g*. Cells were resuspended in 3 mL of red blood cell (RBC) lysis buffer (ACK buffer) for 5 min at room temperature (51). The lysed cells were added to 10 mL of FACS buffer and spun down for 4 min at 400 × *g*. The cells were resuspended in 1 mL radioimmunoprecipitation assay (RIPA) lysis buffer and left at 4°C for 30 min. Cells were spun down at 10,000 × *g* for 5 min at 4°C, and the supernatant was collected. A Bradford assay was performed to determine the protein concentration of the supernatant. Eight hundred micrograms of supernatant was added to 15 μL of Sepharose beads loaded with 5 μg of 18A3 human anti-HA antibody and left overnight rotating at 4°C (52). Samples were then spun down at 2000 × *g* for 5 min and washed 3 times with RIPA buffer. Pelleted beads were then boiled for 10 min and used for Coomassie staining and Western blot assays.

### ELISAs.

Purified influenza virion was diluted to 5 μg/mL and was used to coat the bottom of a Maxisorp 96-well ELISA plate (ThermoFisher). Virus was allowed to bind to the plate overnight at 4°C, and then the plate was blocked with blocking buffer (1% milk in PBS-Tween). Sera was diluted 1:100 in blocking buffer and then serially diluted 3-fold. Plates were incubated with sera for 2 h, after which the plates were washed 3 times with blocking buffer. Plates were incubated with goat α-mouse IgG-HRP secondary antibody (Prometheus) diluted 1:3,000 in blocking buffer and incubated for 1 h. Plates were washed 3 times with blocking buffer and then incubated with 100 μL of SigmaFast OPD substrate for 30 min. Substrate reaction was stopped with 25 μL of 3 M HCl, and absorbance was measured at 450 nm with a Luminometer plate reader (Promega).

### Hemagglutination inhibition assay.

Sera were receptor destroying enzyme (RDE)-treated overnight as previously described and then diluted 2-fold in PBS (53). Virus was diluted to 4 × 10^6^ PFU/mL in PBS–BSA–Pen-Strep, and 25 μL of virus was added to an equal volume of serum in a v-bottom well of a 96-well microtiter plate. Virus and sera were incubated for 1 h at 37°C. Fifty microliters of 0.5% chicken RBCs (Lampire) diluted in PBS was added to each well. Plates were incubated at 4°C for 1 h and then checked for hemagglutination inhibition.

### Statistical analysis and figure graphics.

Student's *t* test statistical analysis was performed with GraphPad Prism 9.2.0 software. Graphs in figures were generated with GraphPad Prism 9.2.0 software, structural images were generated with PyMOL, and BioRender was used to create the mouse graphic in Fig. 4.

### Ethics statement.

The animal study was reviewed and approved by the University of California, Riverside Institutional Animal Care and Use Committee.
