# Supplementary material for: Impact of Protein Nitration on Influenza Virus Infectivity and Immunogenicity
Source: Microbiol Spectr. 2022 Oct 31;10(6):e01902-22. doi: 10.1128/spectrum.01902-22 (PMC9769652; doi:10.1128/spectrum.01902-22)
Supplement: Supplemental file 1 — Fig. S1 to S3. Download spectrum.01902-22-s0001.pdf, PDF file, 0.5 MB [file spectrum.01902-22-s0001.pdf]

Supplemental Material

Impact of Protein Nitration on Influenza Virus Infectivity and Immunogenicity

Dulin H, Hendricks N, Xu D, Gao L, Wuang K, Ai H, Hai R

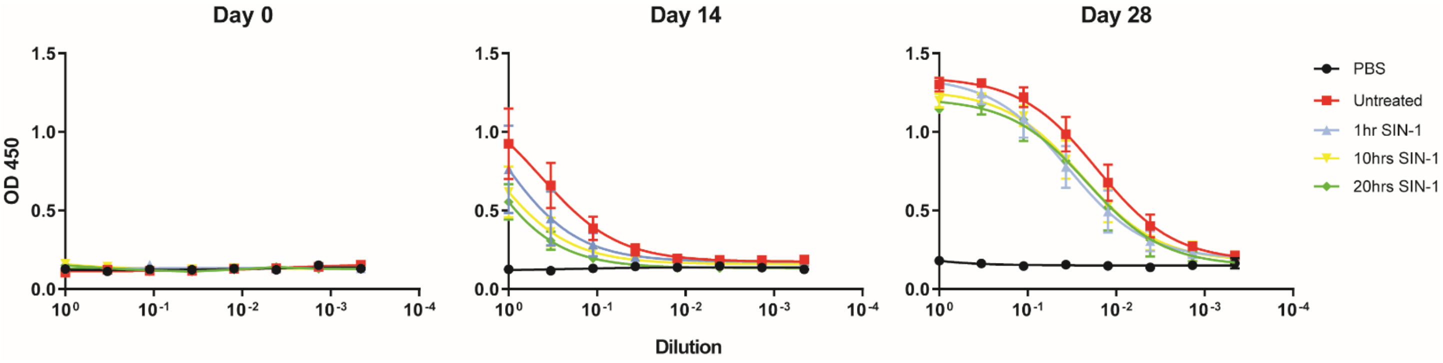

Supplemental Figure 1. Reduced Antibody responses to SIN-1 treated HA protein. Results are for vaccinations with HA protein treated with SIN-1 for 1 hour, 10 hours, or 20 hours. Untreated and 20 hour SIN-1 treated results are displayed in Figure 4B.

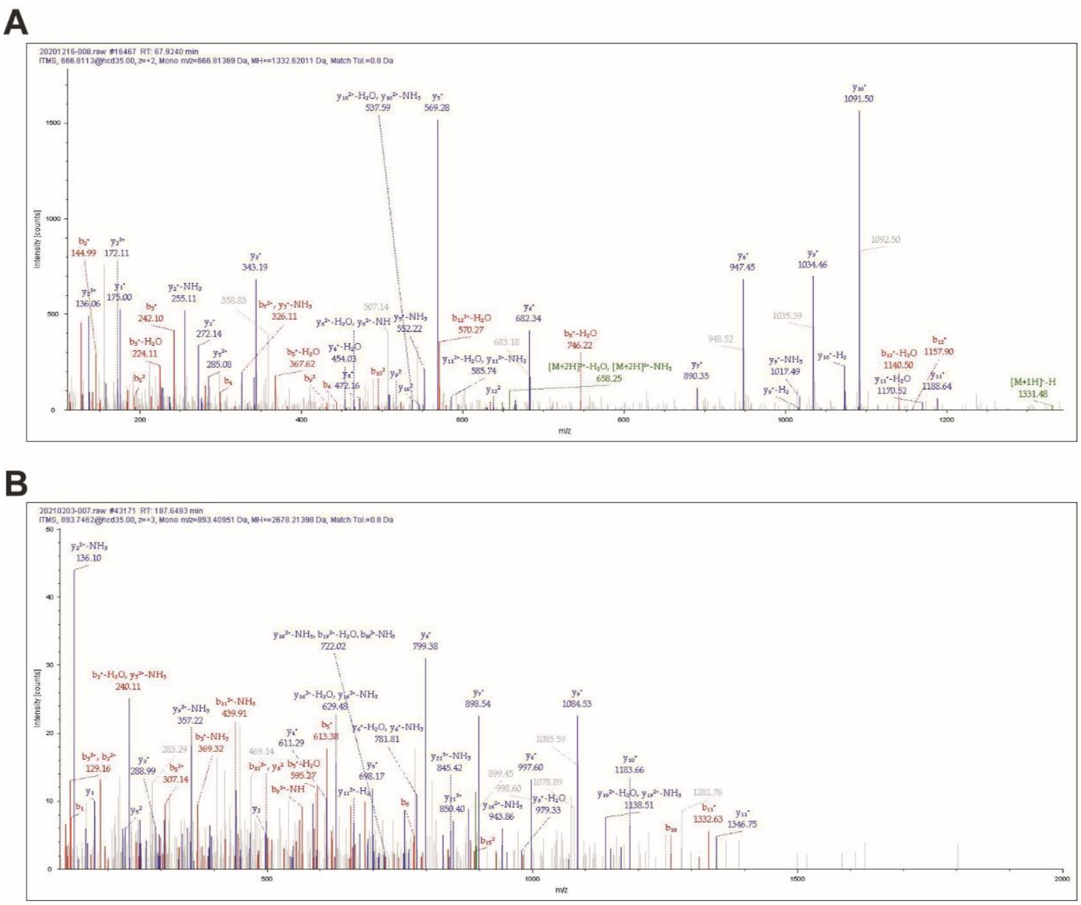

Supplemental Figure 2. (A) Representative MS2 mass spectra of nitrotyrosine-modified purified HA protein peptide GSPGSGY(Nitrotyrosine)IPEAPR. **(B)** Representative MS2 mass spectra of nitrotyrosine modified HA protein from PR8 virion peptide EQQNIYQNENAYVSVVTSNY(Nitrotyrosine)NR. y ions are shown in blue and b ions are shown in red.

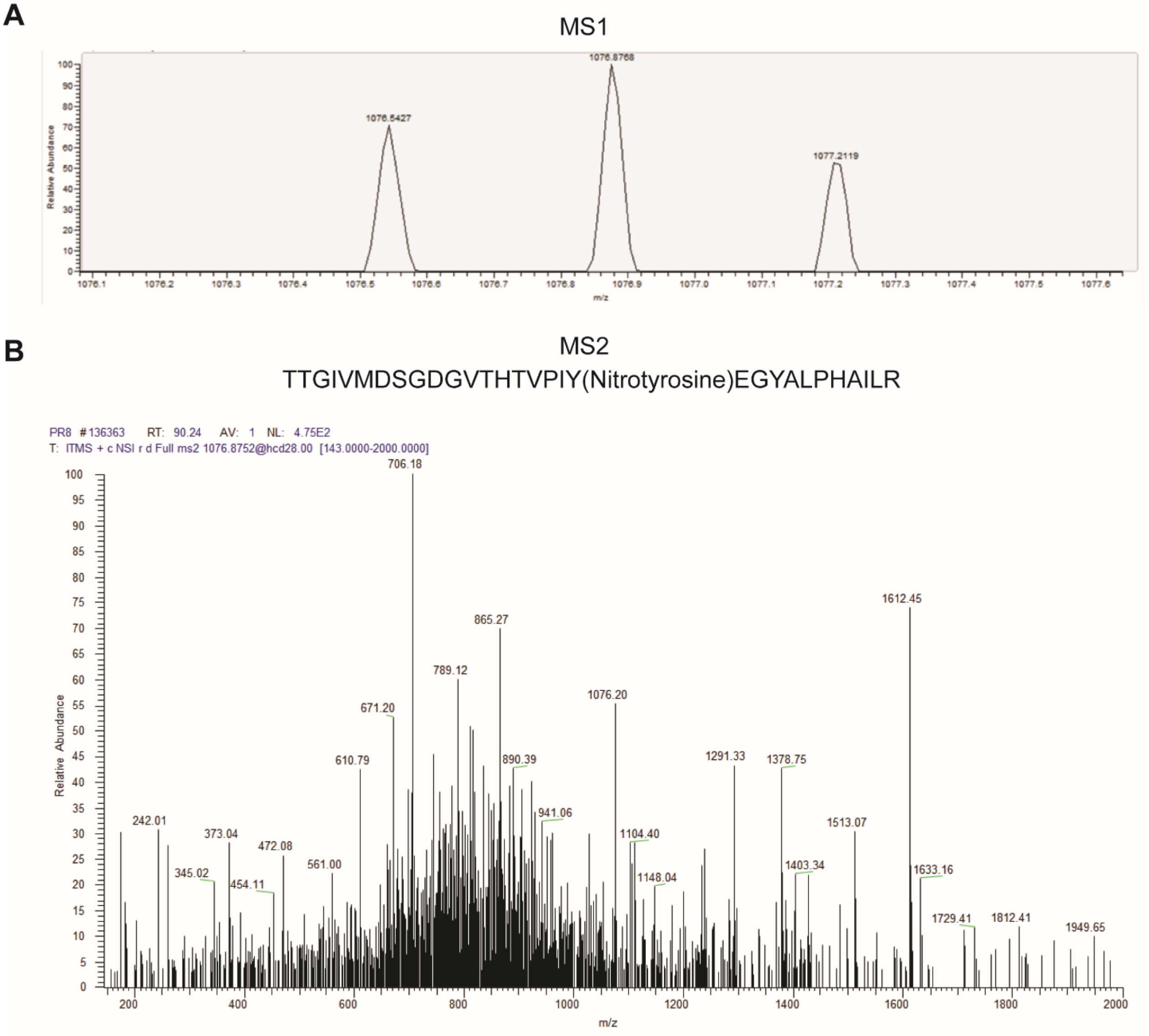

Matches	m/z
y1	175.295
y2	288.14
y4	472.0751
y6	706.1761
y7	819.962
y8	890.389
y9	1053.627
y10	1110.379
y11	1239.334
y6(2+)	354.052
y8(2+)	445.895
y16(2+)	929.061
y25(2+)	1379.476
y26(2+)	1428.893
y28(2+)	1513.999
b2	203.154
b3	260.019
b4	373.0351
b5	472.0751
b13	1234.363
b2-H2O	184.978
b3-H2O	242.01
b5-H2O	454.1051
b17(2+)	834.399
b26(2+)	1379.476

Supplemental Figure 3. MS1 and MS2 peptide spectra matched to nitrotyrosine-modified mouse Actin protein (Protein accession number P63260) from PR8 infected mice, 5 days post infection. MS1 spectra shows peptide peak at 1076.8768 m/z. MS2 spectra is for peptide sequence TTGIVMDSGDGVTHTVPIY(Nitrotyrosine)EGYALPHAILR.
